# Sodium Benzoate Attenuates Secondary Brain Injury by Inhibiting Neuronal Apoptosis and Reducing Mitochondria-Mediated Oxidative Stress in a Rat Model of Intracerebral Hemorrhage: Possible Involvement of DJ-1/Akt/IKK/NFκB Pathway

**DOI:** 10.3389/fnmol.2019.00105

**Published:** 2019-04-30

**Authors:** Weilin Xu, Tao Li, Liansheng Gao, Cameron Lenahan, Jingwei Zheng, Jun Yan, Anwen Shao, Jianmin Zhang

**Affiliations:** ^1^Department of Neurosurgery, Second Affiliated Hospital, School of Medicine, Zhejiang University, Hangzhou, China; ^2^Burrell College of Osteopathic Medicine, Las Cruces, NM, United States; ^3^Department of Neurosurgery, Affiliated Tumor Hospital of Guangxi Medical University, Guangxi Zhuang Autonomous Region, Nanning, China; ^4^Brain Research Institute, Zhejiang University, Hangzhou, China; ^5^Collaborative Innovation Center for Brain Science, Zhejiang University, Hangzhou, China

**Keywords:** DJ-1, sodium benzoate, intracerebral hemorrhage, secondary brain injury neuronal apoptosis, oxidative stress

## Abstract

Intracerebral hemorrhage (ICH) is a devastating disease with high rates of mortality and morbidity. The aim of this study was to explore whether Sodium Benzoate (NaB) could reduce neural cell apoptosis and alleviate neurological deficits after ICH. To assess the therapeutic effects of NaB, first, we measured brain water content, neurobehavior, and blood-brain barrier (BBB) integrity at 24 h after ICH in different groups. Then western blot and immunofluorescence staining (IF) were applied to test the levels of different proteins. Transmission electron microscope (TEM) was used to observe ultra-structures within the cells in different groups. The results showed that levels of DJ-1, p-Akt and p-IκB kinase (IKK) increased after ICH and peaked at 24 h. Besides, NaB significantly upregulated DJ-1 in both cytoplasm and mitochondria, and also increased the levels of p-Akt, p-IKK and Bcl-2/Bax ratio, but decreased the levels of caspase-3 and caspase-9. Additionally, NaB decreased reactive oxygen species (ROS) while increased adenosine triphosphate (ATP), which then improving the neurological functions at 24 h and long-term (21 days) memory and spatial learning ability after ICH. However, the results mentioned above could be greatly reversed by MK2206 and rotenone. Therefore, we concluded that NaB could attenuate secondary brain injury via inhibiting neuronal apoptosis and reducing mitochondria-mediated oxidative stress via DJ-1/Akt/IKK/NFκB pathway.

## Introduction

Intracerebral hemorrhage (ICH), one of the most common types of stroke, is still a serious concern for public health (Feigin et al., 2009). It owns a high rate of disability and death, causing a substantial burden on both patients and their families (van Asch et al., 2010; Krishnamurthi et al., 2014). The incidence rate is rising due to the increasing trend of aging population and the use of anticoagulation or antiplatelet agents (Abdu et al., 2012). The devastating conditions of patients with ICH result from primary mechanical damages and secondary injuries, involving BBB disruption, oxidative stress, neuronal apoptosis and neuro-inflammation (Delcourt et al., 2017; Wu et al., 2017). Although pharmacological treatment and the underlying mechanisms of ICH have been studied for many years, this disease remains a challenge for physicians (Wang et al., 2018).

DJ-1, a homo-dimeric protein, is characterized by multiple biological activities. It was originally identified as an oncogene, and its mutations cause a familial type of Parkinson’s disease-PARK7 (Lill, 2016). Recently, DJ-1 was found to exert its cellular protective effects via anti-oxidative stress. Besides, DJ-1 was reported to protects against mitochondrial damage, signaled by the oxidation of C106 (Canet-Avilés et al., 2004). Thus, DJ-1 is regarded as an endogenous antioxidant in displaying its multiple functions (Yu et al., 2016; Biosa et al., 2017). Regarding the anti-oxidant properties, Tanti found that DJ-1 protected cells from oxidative damage by translocating to mitochondria and preserved the normal functions of mitochondria (Tanti and Goswami, 2014). Additionally, the pro-survival effects of DJ-1 were mediated by activating extracellular signal-regulated kinase (ERK1/2) pathway and phosphatidylinositol-3-kinase (PI3K)/Akt pathway while the anti-apoptotic effects of DJ-1 were mainly mediated by inhibiting ASK1 and mitogen-activated protein kinase kinase kinase 1 (MEKK1/MAP3K1) (Gu et al., 2009; Im et al., 2010; Jaramillo-Gómez et al., 2015; Oh and Mouradian, 2018). Moreover, the neuroprotective effects of DJ-1 were also reported. Yanagisawaa and colleagues demonstrated that administration of recombinant glutathione S-transferase-tagged human DJ-1 could significantly reduce the infarct size in the cerebral ischemic rat model. Additionally, this recombinant human DJ-1 could also reduce H2O2-mediated ROS production in SH-SY5Y cells (Yanagisawa et al., 2008). However, the functions of DJ-1 have not been explored in hemorrhagic stroke. NaB, the sodium salt of an aromatic carboxylic acid, was reported to be a potent agonist of DJ-1 (Khoshnoud et al., 2018). Khasnavis and colleagues found that NaB could upregulate DJ-1, which further exerts neuroprotective effects in a mouse model of Parkinson’s disease (Khasnavis and Pahan, 2014). Therefore, we proposed that NaB could exert its neuroprotective effects in experimental ICH by regulating the expression of DJ-1.

However, the underlying mechanisms of DJ-1-mediated neuroprotective effects are not well understood. Abdel-Aleem GA and colleagues reported that DJ-1 could exert its neuroprotective effects via activation of Akt (Abdel-Aleem et al., 2016). Meanwhile, Aggarwal and Kar both showed that inhibition of Akt phosphorylation could significantly down-regulate the expression of IKK/NF-κB, thereby increasing cellular apoptosis (Aggarwal et al., 2006; Kar et al., 2012). Therefore, we hypothesized that DJ-1 could exert its neuroprotection via the Akt/IKK/NF-κB pathway in the experimental ICH. Moreover, DJ-1 would translocate from the cytoplasm to the outer mitochondrial membrane and exert its anti-oxidative effects to stabilize the homeostasis of mitochondria, which is vital to the normal physiological processes of the neural cells (Tanti and Goswami, 2014).

In this study, we first showed that up-regulation of DJ-1 by NaB could reduce neuronal apoptosis via the Akt/IKK/NFκB pathway and attenuate mitochondria-related oxidative stress in a rat model of ICH.

## Materials and Methods

### Animals

The ethics committee of Zhejiang University approved all of the experimental protocols. All of the experimental steps were conducted based on the NIH. Three hundred and ninety-five rats (male, SD, 280–330 g) were bought from SLAC Laboratory Animal Co., Ltd. (Shanghai, China) and applied in this study.

Animals were housed in a room with 12 h day/night cycle (22 ± 1°C; 60 ± 5% humidity). Animals were offered food and water *ad libitum*.

### ICH Rat Models

We conducted the animal model of ICH based on a previous study (Zheng et al., 2018). Briefly, the rats were placed on a stereotaxic frame after deep anesthetization. Autogenous blood (100 μl) was obtained from the right femoral artery and injected into the right striatum in the position (*x*:*y*:*z* = 3.5:0:5.5 mm) relative to the bregma. (Detailed procedures please see Supplementary Material).

### Behavior Assessment

We used a marking system called the NSS to evaluate neurological function at 24 h after ICH (Zheng et al., 2018). The detailed description of NSS is displayed in Supplementary Table 1. The Long-term neurobehavior assessments were conducted with Morris water maze on each day between the 21st and 25th day after ICH. We performed the water maze test according to a previous report (Detailed procedures please see Supplementary Material; Xie et al., 2018).

### Brain Water Content

The brain water content was assessed at 24 and 72 h after ICH. After receiving deep anesthetization with pentobarbital, the rats were trans-cardial perfused with 0.1 M PBS. The right hemispheres of the rats were quickly collected and weighed (wet weight) after euthanasia. We then put the brains in an oven (105°C) for three days and weighed (dry weight). Finally, the following formula was used to calculate brain water content: [(wet weight - dry weight)/(wet weight)] × 100% (Zheng et al., 2018).

### Evans Blue (EB) Staining

We used EB staining to evaluate the BBB integrity. EB solution (2%, 8 mL/kg, Sigma-Aldrich) was slowly injected via the femoral vein after anesthetization. Two hours later, the injured hemisphere was quickly collected after euthanasia. The sample was homogenized in N, N-dimethylformamide and then incubated in a water bath (50°C) for 48 h and centrifuged at 12,000 × *g* for 30 min. Finally, the supernatant was collected and tested with a spectro-fluorophotometer on the condition that the excitation wavelength and emission wavelength equaled 620 and 680 nm, respectively.

### Immunofluorescence and Calculation of Apoptotic Cells

The rats received euthanasia 24 h after ICH. The brains were processed as previously reported (Zheng et al., 2018). Afterwards, the brains were sliced into 10 mm sections, which were then fixed on slides and used for IF staining. The primary antibodies were DJ-1 (1:250, Abcam ab76008), caspase-3 (1:200, Abcam ab13847), NeuN (1:500, Abcam ab177487). Additionally, TUNEL (Roche Inc., Basel, Switzerland) and caspase-3 staining were applied to quantitatively evaluate the cellular apoptosis. We quantitatively evaluated the neuronal apoptosis by calculating TUNEL and caspase-3 positive cells in five separate fields around the hematoma in three sections at 200 × per brain sample. The finally results were displayed as cells per millimeter (Detailed procedures please see Supplementary Material).

### Measurement of ROS Level

We tested the levels of ROS according to the instructions from a ROS assay kit (JianCheng, China). Perihematomal brain tissues in ICH groups and corresponding area in sham group were used for testing ROS. The detailed information has been reported in our recent study (Zheng et al., 2018; Detailed procedures please see Supplementary Material).

### Measurement of ATP Levels

We tested the level of ATP according to the instructions from an ATP assay kit (Beyotime, Shanghai, China). Perihematomal brain tissues in ICH groups and corresponding area in sham group were used for ATP evaluation. The detailed information has been reported in our recent study (Zheng et al., 2018; Detailed procedures please see Supplementary Material).

### Drug Administration

The NaB was purchased from Sigma. This drug was dissolved in sterile saline and intraperitoneally given at 1 h after ICH. The MK2206 was dissolved in sterile saline and intracerebroventricularly given at 1 h after ICH. DJ-1 siRNA was purchased from Thermo Fisher Scientific company. Five hundred pmol siRNA dissolved in 10 μl sterile water was intracerebroventricularly administrated at 48 h before ICH, as previously reported (Zheng et al., 2018).

### Western Blot Analysis

Perihematomal brain tissues in ICH groups and corresponding area in sham group were collected for western blot anslysis. We extracted proteins of mitochondria, according to the manufacturer’s protocol from Isolation Kit for Tissue protocol (Pierce Biotechnology, Rockford, IL, United States). The detailed procedures of western was reported in one of our previous study.31 The primary antibodies were listed as follows: DJ-1 (1:15000, Abcam ab76008), p-Akt (1:2000, CST 4060s), Akt (1:3000, CST 4691), p-IKK (1:1000, CST 2697), IKK (1:1000, CST 2682), NF-κB p65 (1:3000, ab86299), Bax (1:1000, Abcam ab32503), Bcl-2 (1:500, Abcam ab59348), caspase-3 (1:500, Abcam ab13847), cleaved caspase-9 (1:1500, Abcam ab25758) and β-actin (1:5000, Abcam ab8226), NDUFS8 (1:2000, Abcam ab180183), COX IV (1:1000, CST 4844).

### Transmission Electron Microscopy (TEM)

We performed TEM, as previously reported (Zheng et al., 2018). Briefly, after euthanasia, the brain tissues around hematoma in ICH groups and corresponding area in sham group of the rats were collected and further processed into 100nm sections, which were then stained with 4% uranyl acetate (20 min) and 0.5% lead citrate (5 min). Finally, the sections were visualized using the TEM (Philiphs Tecnai 10).

### Experimental Design (Figure 1)

**Figure 1:**
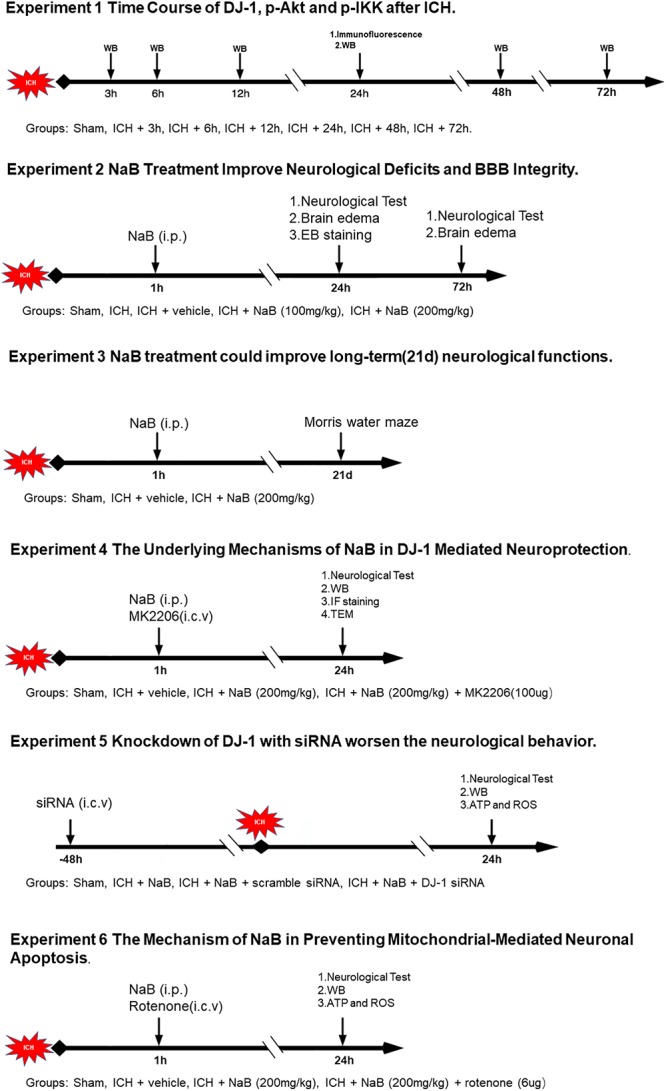
Experimental design and animal groups.

The study was divided into six parts, a total of 395 animals were used in this study, including 12 dead animals. Ninety-one rats were in Sham group and 304 rats were in ICH group. The mortality was almost 4% (12/304; Supplementary Table 2).

In experiment 1, forty-six rats were divided randomly into seven groups (sham, 3, 6, 12, 24, 48, and 72 h, *n* = 6) for western blot. An additional four rats in the sham and ICH (24 h) groups were used for IF staining.

In experiment 2, ninety rats were divided into five groups: sham (*n* = 18), ICH (*n* = 18), ICH + vehicle (*n* = 18), ICH + NaB (100 mg/kg, *n* = 18) and ICH + NaB (200 mg/kg, *n* = 18). Neurobehavior and brain edema were evaluated at 24 and 72 h after ICH in each group.

In experiment 3, to explore the effects of treatment with NaB (NaB, Sigma) on long-term spatial learning and memory abilities, thirty rats were separated into three groups: sham (*n* = 10), ICH + vehicle (*n* = 10), and ICH + NaB (200 mg/kg, *n* = 10). We performed Morris water maze each day between the 21st and 25th day after ICH.

In experiment 4, fifty-three rats were distributed randomly into four groups: sham (*n* = 14), ICH + vehicle (*n* = 14), ICH + NaB (200 mg/kg, *n* = 14), or ICH + NaB (200 mg/kg) + MK2206 (100 μg, *n* = 11, Selleck Chemicals, Houston, TX). MK2206 and NaB were both administered 1 h after ICH. They were applied intracerebroventricularly and intraperitoneally, respectively. Western blot analysis (*n* = 6), TEM (*n* = 3) and IF staining of TUNEL and caspase-3 (*n* = 5) were applied to explore the underlying mechanisms.

In experiment 5, we divided seventy-two rats into four groups: sham (*n* = 18), ICH + NaB (*n* = 18), ICH + NaB + scramble siRNA (500 pmol, Thermo, *n* = 18), and ICH + NaB + DJ-1 siRNA (500 pmol, Thermo, *n* = 18). DJ-1 siRNA was intracerebroventricularly injected at 48 h before ICH induction. Six rats per group were utilized in the western blot analysis, ATP level and ROS level, respectively.

In experiment 6, we used rotenone (Sigma-Aldrich, St. Louis, MO, United States) to verify whether DJ-1 could exert its neuroprotective effects via preventing mitochondrial-mediated apoptosis in the model of ICH. Ninety-two rats were separated into four groups: sham (*n* = 23), ICH + vehicle (*n* = 23), ICH + NaB (200 mg/kg, *n* = 23), or ICH+ NaB (200 mg/kg) + rotenone (6 μg, *n* = 23). Six rats from each group were used for western blot analysis, ATP level and ROS level, respectively. IF staining of TUNEL and caspase-3 was also performed to test the neuroprotective effects of DJ-1.

### Statistical Analysis

We displayed the data as mean ± SD. SPSS 22.0 software was applied to analyze the data (IBM, United States). First, we tested the data normality using the Shapiro–Wilk normality test. If the data met the requirements of satisfied normality and homogeneity of variance, one-way ANOVA was applied for the comparison between the different groups. For the data that failed the normality test, non-parametric statistics were applied, with *P* < 0.05 indicating statistical significance.

## Results

### Temporal Patterns of DJ-1, p-Akt and p-IKK After ICH

**Figure 2:**
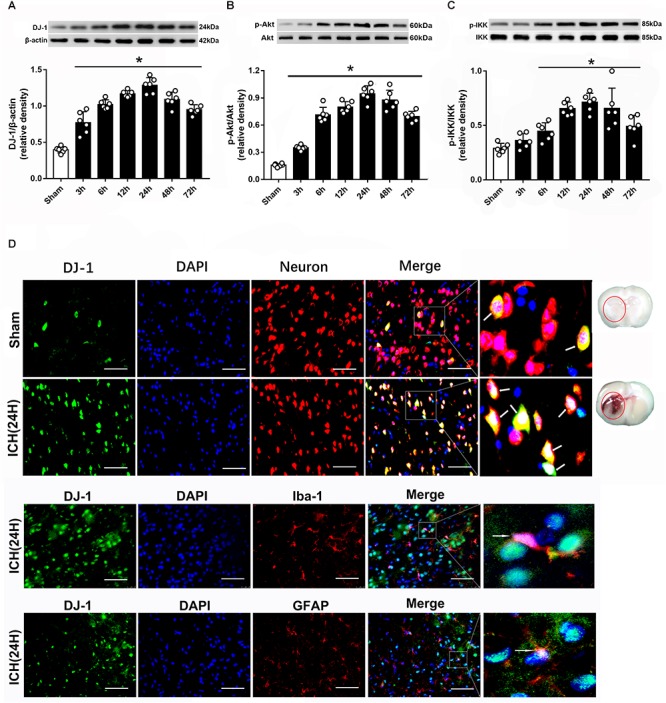
Expression of DJ-1, pAkt and pIKK is increased upon ICH. **(A)** Time course of DJ-1 in injured hemisphere after ICH; **(B)** Time course of p-Akt; **(C)** Time course of p-IKK; *n* = 6 for each group. The bars represent the mean ± SD. ^∗^*p* < 0.05 vs. sham; **(D)** Representative microphotographs of immunofluorescence staining showing localization of DJ-1 (green) and NeuN (neuron), Iba-1 (microglia) and GFAP (astrocyte) in the perihematomal region after ICH (*n* = 2 for each group). Scale bar = 50 μm.

Western blot was used to evaluated the protein level of DJ-1, p-Akt, and p-IKK at 3, 6, 12, 24, 48 and 72 h after ICH. The results showed that DJ-1 significantly increased at 3 h and peaked at 24 h after ICH (*P* < 0.05, Figure 2A). Additionally, p-Akt and p-IKK levels were upregulated at 6 h and reached a peak at 24 h after ICH (*P* < 0.05, Figure 2B,C). Moreover, IF staining suggested that DJ-1 was colocalized with neuron, microglia and astrocytes and the level of DJ-1 was dramatically upregulated after ICH (Figure 2D).

### NaB Improved Short-Term Neurobehavior and Reduced Brain Edema After ICH

**Figure 3:**
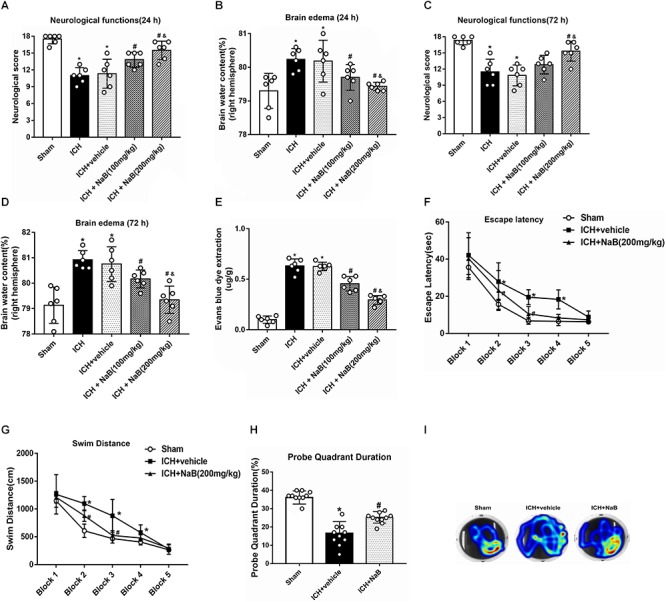
NaB prevents short- and long-term neurological functions, brain edema and BBB leakage upon ICH. **(A,C)** The quantification of neurological functions at 24 h and 72 h after ICH; **(B,D)** The quantification of brain water content at 24 and 72 h after ICH; **(E)** The quantification of Evans blue dye extravasation at 24 after ICH (*n* = 6 for each group). The effects of NaB on long-term neurobehavioral outcomes after ICH. **(F)** Escape latency and **(G)** swim distance of Morris water maze on days 21 to 25 after ICH; **(H)** Probe quadrant duration of Morris water maze on day 25 after ICH; **(I)** The representative images of Morris water maze trials. The bars represent the mean ± SD. ^∗^*p* < 0.05 vs. sham, ^#^*p* < 0.05 vs. ICH + vehicle at 24 h, ^&^*p* < 0.05 vs. ICH + NaB (100 mg/kg), *n* = 10 per group.

Two different dosages of NaB (100 and 200 mg/kg) were intraperitoneally injected at 1 h after ICH. In order to comprehensively evaluate the effects of NaB, we tested the neurological functions and assessed the brain edema at 24 and 72 h after ICH. The results indicated that the rats in ICH group had significant neurobehavioral impairment compared with that of the sham group in both 24 and 72 h after ICH (*P* < 0.05, Figure 3A,C). NaB treatment notably increased the neurological score in the ICH + NaB group. Moreover, the higher dosage of NaB (200 mg/kg) was shown to be more effective in alleviating neurological deficits (*P* < 0.05, Figure 3A,C).

The rats that received autogenous blood injection developed significant brain edema in 24 h and up to 72 h after ICH compared to the rats in sham group (*P* < 0.05, Figure 3B,D). The higher dosage of NaB (200 mg/kg) was more effective in reducing brain edema than low dosage (100 mg/kg; *P* < 0.05, Figure 3B,D). Additionally, EB leakage was significantly increased in the ICH group, which was notably reduced with higher NaB treatment (*P* < 0.05, Figure 3E). Therefore, we chose to use the higher dosage of NaB in both the long-term outcome and mechanism studies.

### Administration of NaB Improved Long-Term Neurobehavior After ICH

The results of escape latency and swim distance indicated that the rats in the ICH + vehicle group had more difficulty finding the submerged platform than those in the sham group (*P* < 0.05, Figure 3F,G). After the treatment with NaB, the time to find the platform decreased on blocks 3 to 4 and swim distance was also reduced on block 4 (*P* < 0.05, Figure 3F,G). Regarding the probe quadrant trial, the rats in ICH + vehicle resided in the target quadrant for a shorter time when compared to the rats in the sham group. However, the rats in ICH + NaB group resided longer time than the rats in the ICH + vehicle group (*P* < 0.05, Figure 3H,I).

### Ultrastructure Changes of Brain Tissues After ICH

**Figure 4:**
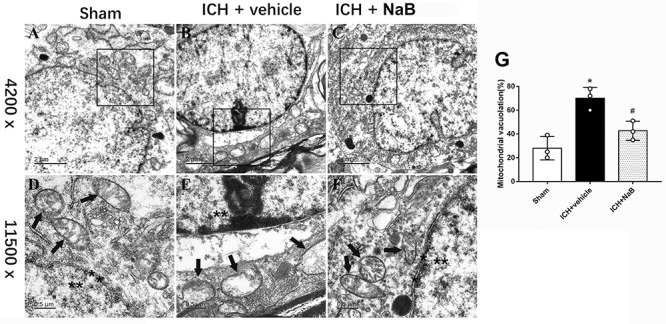
Transmission electron microscopy images of the morphometric changes of brain tissues. **(A)** Sham; **(B)** ICH + vehicle; **(C)** ICH + NaB (scar bar = 2 μm); **(D–F)** Magnification of **(A–C)** (scar bar = 0.5 μm); **(G)** The quantification of mitochondrial vacuolation rate. ^∗^*p* < 0.05 vs. sham, ^#^*p* < 0.05 vs. ICH + vehicle at 24 h, *n* = 3 per group. Mitochondria (black arrows) with prominent cristae and an intact membrane and nucleus (asterisk) with a clear membrane and homogenous chromatin was observed in sham group. The induction of ICH results in irregular mitochondria with damaged cristae and condensed chromatin. However, NaB administration notably reversed the results.

We used TEM to observe the ultrastructural changes within the brain tissues after being subjected to ICH. Under normal conditions, we are able to see the mitochondria with prominent cristae and an intact membrane (Figure 4A,D). The nucleus in the sham group showed a clear membrane and homogenous chromatin. After the induction of ICH, irregular mitochondria with ruptured membranes and condensed chromatin were observed (Figure 4B,E). However, NaB administration notably reversed the results (Figure 4C,F). Besides, the quantification of mitochondrial vacuolation between different groups indicated that NaB treatment significantly improved the integrity status of mitochondria (*P* < 0.05, Figure 4G).

### Administration of NaB Reduced Oxidative Stress and Improved the Functions of Mitochondria

**Figure 5:**
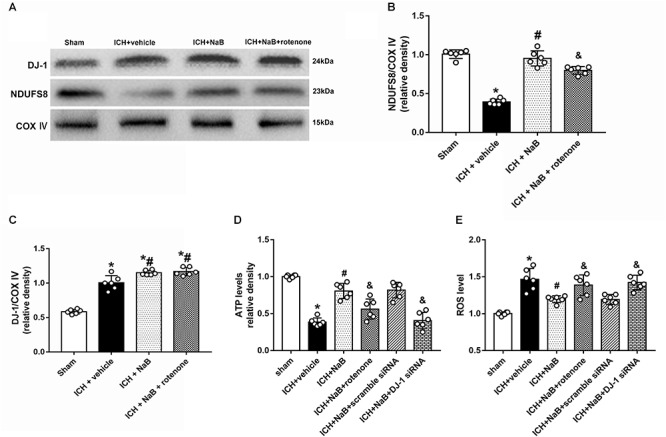
Intraperitoneal administration of NaB protects mitochondria via anti-oxidative stress at 24 h after ICH. **(A)** Representative Western blot images. Quantitative analyses of **(B)** NDUFS8 and **(C)** DJ-1 levels in mitochondria. **(D)** Level of ATP at 24 h after ICH; **(E)** Level of ROS at 24 h after ICH. *n* = 6 for each group. The bars represent the mean ± SD. ^∗^*p* < 0.05 vs. sham, ^#^*p* < 0.05 vs. ICH + vehicle, ^&^*p* < 0.05 vs. ICH + NaB.

NADH: ubiquinone oxidoreductase core subunit S8 was always used to indicate the level of complex I.^25^ (Zhou et al., 2015). We extracted the proteins from mitochondrial fractions. The results showed that mitochondrial NDUFS8 and ATP levels notably decreased at 24 h after ICH (*P* < 0.05 vs. sham group, Figure 5A,B,D). Meanwhile, the levels of mitochondrial DJ-1 and ROS significantly increased in the ICH + vehicle (*P* < 0.05 vs. sham group, Figure 5C,E). However, all of the results were reversed by the administration of NaB.

### Administration of NaB Reduced Mitochondrial-Mediated Apoptosis After ICH

**Figure 6:**
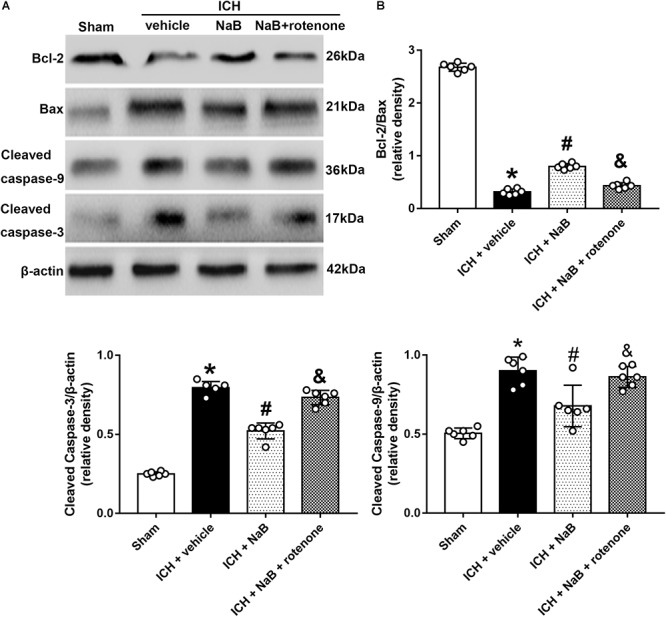
Intraperitoneal administration of NaB reduces mitochondrial related apoptosis at 24 h after ICH, which could be partly reversed by rotenone. **(A)** Representative Western blot images. **(B)** Quantitative analyses of Bcl-2, Bax, Cleaved Caspase-3 and caspase-9. *n* = 6 for each group. The bars represent the mean ± SD. ^∗^*p* < 0.05 vs. sham, ^#^*p* < 0.05 vs. ICH + vehicle, ^&^*p* < 0.05 vs. ICH + NaB.

In an effort to verify whether DJ-1 could exert its neuroprotection via prevention of mitochondrial-mediated apoptosis in the model of ICH, we used rotenone, an inhibitor of complex I, together with NaB. The results indicated that NaB treatment notably upregulated mitochondrial DJ-1 in the mitochondria (Figure 5C), as well as the anti-apoptotic factor, such as Bcl-2, but decreased the level of pro-apoptotic factors, Bax, cleaved caspase-3 and cleaved caspase-9 (*P* < 0.05, Figure 6A,B). However, the administration of rotenone significantly reversed the results induced by DJ-1 and NaB (*P* < 0.05).

### Neuroprotective Effects of DJ-1 Act via the Akt/IKK/NF-κB Pathway

**Figure 7:**
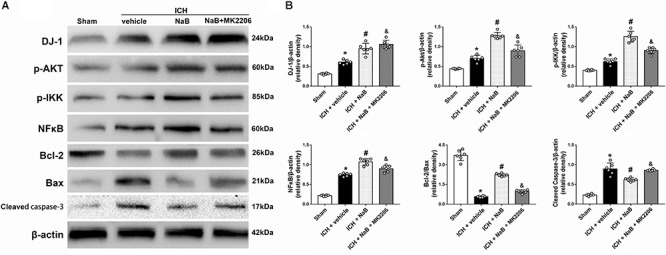
Intraperitoneal administration of NaB partially prevents the molecular changes induced by ICH at 24 h after ICH. **(A)** Representative Western blot images. **(B)** Quantitative analyses of DJ-1, p-Akt, p-IKK, NFκB, Bcl-2, Bax and Cleaved Caspase-3; *n* = 6 for each group. The bars represent the mean ± SD. ^∗^*p* < 0.05 vs. sham, ^#^*p* < 0.05 vs. ICH + vehicle, ^&^*p* < 0.05 vs. ICH + NaB.

**Figure 8:**
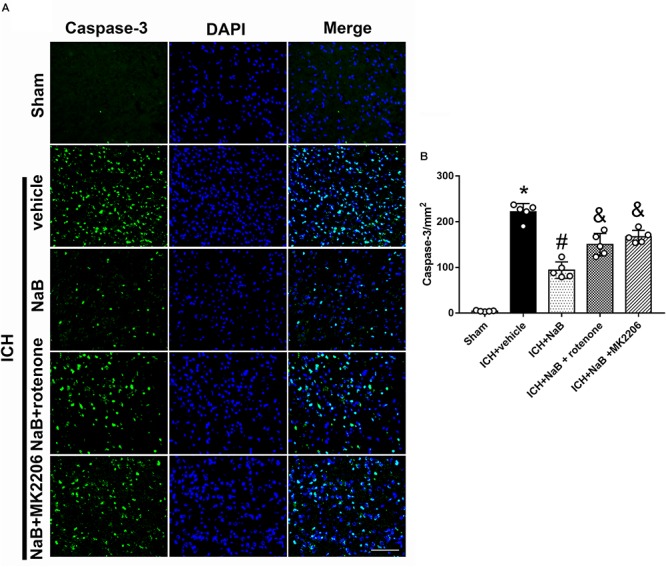
The administration of NaB significantly decreased the number of Caspase-3 and DAPI double-stained cells in the perihematomal region 24 h after ICH. **(A)** Representative microphotographs showed the co-localization of DAPI (blue) with Caspase-3 (green)-positive cells in injured brain hemisphere at 24 h after ICH; **(B)** Quantitative analysis of Caspase-3 positive cells showed that NaB decreased the number of apoptotic cells after ICH. The bars represent the mean ± SD. Scale bar = 100 μm. *n* = 5. ^∗^*p* < 0.05 vs. sham, ^#^*p* < 0.05 vs. ICH + vehicle, ^&^*p* < 0.05 vs. ICH + NaB.

**Figure 9:**
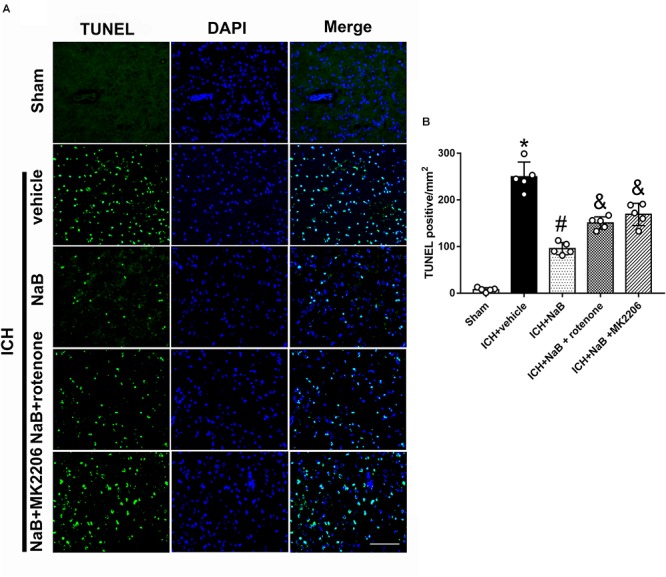
The administration of NaB significantly decreased the number of TUNEL and DAPI double-stained cells in the perihematomal region 24 h after ICH. **(A)** Representative microphotographs showed the co-localization of DAPI (blue) with TUNEL (green)-positive cells in injured brain hemisphere at 24 h after ICH; **(B)** Quantitative analysis of TUNEL-positive cells showed that NaB decreased the number of apoptotic cells after ICH. Scale bar = 100 μm. *n* = 5. ^∗^*p* < 0.05 vs. sham, ^#^*p* < 0.05 vs. ICH + vehicle, ^&^*p* < 0.05 vs. ICH + NaB.

In order to verify whether DJ-1 exerted its neuroprotective effects via Akt/IKK/ NF-κB pathway, MK2206, a specific inhibitor of Akt, was intracerebroventricularly injected 1 h after ICH. The use of MK2206 had no effect on the level of DJ-1, which was upregulated after ICH (*P* < 0.05, Figure 7A,B). While NaB upregulated the levels of p-Akt, p-IKK, and NF-κB, we found that MK2206 had the opposite effect with significant reduction (*P* < 0.05 vs. ICH + NaB). Moreover, the administration of NaB increased the Bcl-2/Bax ratio while simultaneously reducing the levels of cleaved caspase-3, thereby leading to a reduction in cellular apoptosis. However, MK2206 greatly suppressed these neuroprotective effects (*P* < 0.05, Figure 7A,B). Besides, the IF staining of TUNEL and caspase-3 indicated that TUNEL and caspase-3 positive cells significantly increased after ICH (*P* < 0.05, ICH vs. sham, Figure 8, 9). However, NaB treatment could reverse these results (*P* < 0.05, ICH + vehicle, Figure 8, 9).

### Assessment of the Depletion Efficiency of DJ-1 siRNA With Naïve Rats

In order to test the depletion efficiency of DJ-1 siRNA, we applied DJ-1 siRNA in naïve animals. The results showed that DJ-1 siRNA decreased the level of DJ-1 by 38.7% on average (Supplementary Figure 1).

### Selective Knock-Down of DJ-1 With siRNA Increased Neuronal Apoptosis 24 h After ICH

**Figure 10:**
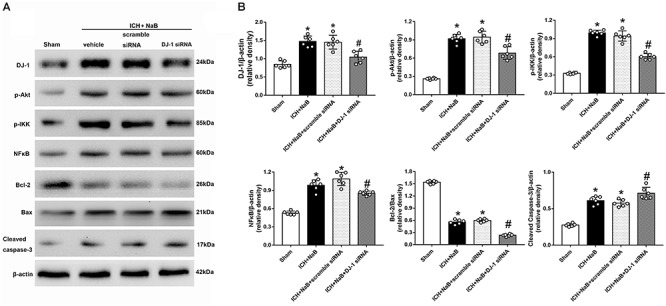
Knockout of DJ-1 with siRNA inhibited NaB mediated anti-apoptotic pathway and related protein changes after ICH. **(A)** Representative Western blot images. **(B)** Quantitative analyses of DJ-1, p-Akt, p-IKK, NFκB, Bcl-2, Bax and Cleaved Caspase-3. *n* = 6 for each group. The bars represent the mean ± SD. ^∗^*p* < 0.05 vs. sham, ^#^*p* < 0.05 vs. ICH + vehicle.

We used DJ-1 siRNA to prove the neuroprotective effects of DJ-1. DJ-1 siRNA or scramble siRNA was intracerebroventricularly administrated at 48 h before ICH. Western blot analysis indicated that the protein level of DJ-1 significantly decreased after the use of DJ-1 siRNA, as well as its downstream targets, p-Akt, p-IKK (*P* < 0.05 ICH + vehicle, Figure 10A,B). Additionally, the use of DJ-1 siRNA significantly increased caspase-3 level while decrease Bcl-2/Bax ratio (*P* < 0.05).

## Discussion

In this study, we explored the neuroprotective effects of NaB through a new mechanism mediated by DJ-1/Akt/IKK/ NF-κB pathway in the experimental ICH in rats. The novel findings in this study indicate that: (1) the expression of DJ-1, along with its downstream effectors, Akt and IKK was upregulated and peaked at 24h after ICH; the DJ-1 was widely expressed in the neurons; (2) the administration of NaB significantly improved short- and long-term neurobehavior, defended BBB integrity and reduced both brain edema and cellular apoptosis by up-regulation of DJ-1, and its downstream targets (Akt, IKK, NF-κB, Bcl-2), whereas reducing Bax, caspase-9 and caspase-3 expression; (3) knockdown of DJ-1 with specific siRNA significantly increased the pro-apoptotic proteins and ROS level while reducing the anti-apoptotic factors and ATP level, which finally worsen the neurological functions and brain edema; (4) the use of MK2206 also abolished the neuroprotective effects of DJ-1 induced by NaB; (5) the increased level of DJ-1 induced by NaB notably reduced ROS level and increased ATP level, thus further preventing mitochondria-mediated apoptosis by protecting complex I, which could be reversed by the use of rotenone.

ICH usually occurs when small vessels rupture due to long-standing hypertension (Chu and Sansing, 2017). Direct damage from blood accumulation and secondary injuries makes ICH a devastating disease characterized by high morbidity and mortality. The oxidative stress, which is primarily caused by an imbalance between pro-oxidants (ROS/RNS) and anti-oxidants, was reported to contribute greatly to the pathogenesis of ICH (Xie et al., 2017; Zeng et al., 2017). Therefore, scavenging excessive free radicals and ROS will be beneficial to preventing brain injury after ICH. DJ-1, an endogenous antioxidant, has attracted great attention, especially for its effects in the central nervous system (CNS). Daijiro Yanagisawaa and his colleagues demonstrated that the administration of recombinant human DJ-1 could significantly decrease infarct size in their cerebral ischemic rat model by reducing ROS-mediated neuronal injury (Yanagisawa et al., 2008). The neuroprotective effects of DJ-1 was largely mediated via inactivation of PTEN while activation of PI3K/Akt pathway (Choi et al., 2014). Besides, Kaneko and colleagues found that DJ-1 translocated to the healthy mitochondria immediately after stroke and exerted neuroprotective and/or neurorestorative effects (Kaneko et al., 2014). Moreover, DJ-1 was also reported to alleviate the spatial learning and memory impairment in a mouse model of Alzheimer’s Disease (Kitamura et al., 2017). In this study, we explored levels of DJ-1, p-Akt, and p-IKK at different time-points after ICH. The results indicated that DJ-1, p-Akt, and p-IKK notably increased and peaked at 24 h after ICH. The results above suggested that DJ-1 was significantly involved in the suppression of ROS production and promotion of anti-apoptotic factors in the experimental ICH model.

NaB could increase the level of DJ-1 and was used to study the functions of DJ-1. NaB, a widely used food preservative, was reported to display strong anti-oxidative stress properties (Nair, 2001). Additionally, Toth and colleagues reported that NaB was very safe and that the mice in their study did not exhibit any noticeable side effects, even though they received NaB (2%) for their entire lives (Toth, 1984).

Moreover, Saurabh and colleagues reported that DJ-1 was upregulated by NaB treatment, possibly via regulation of the mevalonate pathway (Khasnavis and Pahan, 2014). In this study, we found that DJ-1 levels were upregulated by NaB in a typical dose-dependent manner. NaB treatment significantly increased the levels of DJ-1, NF-κB, and Bcl-2, whereas it decreased Bax and caspase-3 levels. These neuroprotection effects were significantly offset by DJ-1 siRNA. All of the results abovementioned suggested that the NaB exerted its anti-oxidative and anti-apoptotic effects via up-regulation of DJ-1. Additionally, we also investigated the role of DJ-1 in the mitochondria-mediated apoptosis. DJ-1, a potent anti-oxidant, was reported to translocate to the mitochondria and directly protect complex I by scavenging the excessive ROS and free radicals (Heo et al., 2012). In the present study, NDUFS8 and ATP levels were significantly decreased whereas ROS, cleaved caspase-3, and cleaved caspase-9 levels were increased 24 h after the ICH. The administration of NaB significantly up-regulate the expression of DJ-1 and the level of NDUFS8 and ATP, but reduce the levels of ROS, cleaved caspase-3, cleaved caspase-9. However, this protection provided from the up-regulation of DJ-1 was dramatically offset by rotenone. All of the results indicated that DJ-1 prevented mitochondria-mediated apoptosis by protecting complex I.

**Figure 11:**
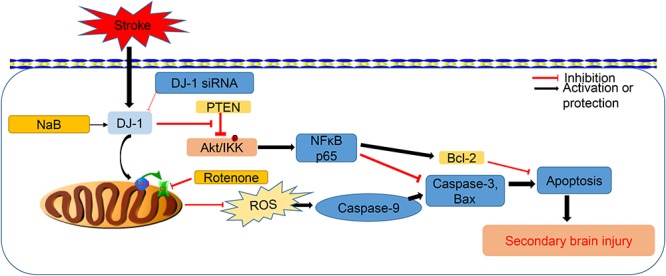
The schematic pathway for describing how DJ-1 mediated anti-apoptotic effects after ICH.

In order to prove whether the neuroprotective effects of DJ-1 were mediated via Akt/IKK/NFκB pathway, MK2206, a highly selective Akt inhibitor, was intracerebroventricularly injected at 1 h after ICH. Akt plays a major role in the regulation of cell growth and survival (Paraskevopoulou and Tsichlis, 2017). It is activated via phosphorylation by PI3K, but can be inhibited by activation of the tumor suppressor PTEN (Qin et al., 2018; Zhang and Cui, 2018). However, DJ-1 was reported to directly interact with PTEN and suppress its phosphorylation ability (Choi et al., 2014), which promotes the activation of Akt from PI3k. In the present study, DJ-1 and p-Akt levels were notably upregulated and peaked at 24 h after ICH. Moreover, NaB treatment would further promote phosphorylation of Akt, the results of which would activate its downstream targets, including IKK and NFκB. However, these neuroprotective effects of DJ-1 and NaB were significantly offset by DJ-1 siRNA. Under normal conditions, IkB suppresses the activation of NF-κB. Once IkBa was degraded from phosphorylation by IKK, NF-κB would translocate to the nucleus and promote the expression of its target genes, which could further facilitate anti-apoptosis and promote cell proliferation (DiDonato et al., 2012). In this study, IKKβ, NFκB, p65, and Bcl-2 were significantly up-regulated after the administration of NaB while Bax and caspase-3 levels were reduced. However, these neuroprotective effects of NaB were significantly offset by the Akt inhibitor, MK2206. Based on the results above, we were able to conclude that NaB exerts its neuroprotective effects via DJ-1/Akt/IKKβ/NFκB pathway in a rat model of ICH. Overall, the neuroprotective effects of NaB were conducted by two different ways, namely, Akt/IKK/NFκB activation and mitochondrial protection, both of which notably reduced neuronal apoptosis (Figure 11). The only connection of these two mechanisms was DJ-1. However, in this study, we only focused on the anti-apoptotic effects of DJ-1 without further explore the relationship these two mechanisms mediated by DJ-1.

There were some limitations of this study. First, neuroprotection of NaB was verified in many ways, but only its role in DJ-1/Akt/IKK/NFκB pathway was investigated in this study. Second, although NaB mediated neuroprotective effects via upregulating the expression of DJ-1, the underlying mechanisms explaining how NaB increases the level of DJ-1 are still unclear. Moreover, the mechanism detailing how NaB promotes DJ-1 to translocate from the cytoplasm to mitochondria has not been thoroughly explored either.

## Ethics Statement

The ethics committee of Zhejiang University approved all of the experimental protocols. All of the experimental steps were conducted based on the NIH.

## Author Contributions

WX and LG designed the study. WX, LG, and TL completed the experiments. AS and LG performed statistical analysis. WX and JiaZ wrote the manuscript. CL and WX revised the manuscript. WX, TL, and JY finished the revision. JinZ and AS participated in discussion development and provided expert guidance.

## Conflict of Interest Statement

The authors declare that the research was conducted in the absence of any commercial or financial relationships that could be construed as a potential conflict of interest.

## Abbreviations

ASK1: apoptosis signal-regulating kinase 1
ATP: adenosine triphosphate
BBB: blood brain barrier
EB: Evans Blue
ERK: extracellular regulated protein kinases
ICH: intracerebral hemorrhage
IKK: IκB kinase
IKK: inhibitor of nuclear factor kappa-B kinase
NaB: sodium benzoate
NDUFS8: NADH: ubiquinone oxidoreductase core subunit S8
NSS: neurological severity score
PBS: phosphate buffer saline
PTEN: phosphatase and tensin homolog
ROS: reactive oxygen species
RT-PCR: reverse transcription-polymerase chain reaction
SD: Sprague–Dawley
TEM: transmission electron microscopy
TUNEL: terminal deoxynucleotidyl transferase (TdT)-mediated dUTP nick end labeling.
