# Supplementary material for: Sodium Benzoate Attenuates Secondary Brain Injury by Inhibiting Neuronal Apoptosis and Reducing Mitochondria-Mediated Oxidative Stress in a Rat Model of Intracerebral Hemorrhage: Possible Involvement of DJ-1/Akt/IKK/NFκB Pathway
Source: Front Mol Neurosci. 2019 Apr 30;12:105. doi: 10.3389/fnmol.2019.00105 (PMC6503040; doi:10.3389/fnmol.2019.00105)
Supplement: Supplementary file 1 [file Data_Sheet_1.docx]

| **Supplemental Table 1. Neurological Severity Scores (NSS)** | | |
| --- | --- | --- |
|  | **Items** | **Score** |
| **raising rat by tail (normal=0; maximum=3)** | | (3) |
|  | flexion of forelimb | 1 |
|  | flexion of hindlimb | 1 |
|  | head moved >10° to vertical axis within 30s | 1 |
|  | placing rat on floor (normal; maximum=3) | (3) |
|  | normal walk | 0 |
|  | inability to walk straight | 1 |
|  | circling toward paretic side | 2 |
|  | falls down to paretic side | 3 |
| **sensory tests (normal=0; maximum=2)** | | (2) |
|  | placing test (visual and tactile test) | 1 |
|  | proprioceptive test (deep sensation) | 1 |
| **beam balance tests (normal=0; maximum=6)** | | (6) |
|  | balances with steady posture | 0 |
|  | grasps side of beam | 1 |
|  | hugs beam and 1 limb falls down from beam | 2 |
|  | hugs beam and 2 limbs falls down from beam, or spins on beam(>60s) | 3 |
|  | attempts to balance on beam but falls off(>40s) | 4 |
|  | attempts to balance on beam but falls off(>60s) | 5 |
|  | falls off; no attempt to balance or hang on to beam(<20s) | 6 |
| **reflex absence and abnormal movements** | | (4) |
|  | pinna reflex (head shake when auditory meatus is touched) | 1 |
|  | corneal reflex (eye blink when cornea is lightly touched with cotton) | 1 |
|  | startle reflex (motor response to a brief noise) | 1 |
|  | seizure, myoclonus, myodystony | 1 |
| **maximum points** | | (18) |

**Supplemental table 2**. The animal usage.

| Part | Groups | Long term neurological test | Brain Edema | ATP and ROS | TEM | Western Blot | IF | dead | Sum |
| --- | --- | --- | --- | --- | --- | --- | --- | --- | --- |
| Exp. 1 | Sham | - | - | - | - | 6 | 2 | 12 | 395 |
|  | ICH (3, 6, 12, 24, 48h, 72 h) | - | - | - | - | 36 | 2 |  |  |
| Exp. 2 | Sham | - | 12+6 (EB) | - | - | - | - |  |  |
|  | ICH | - | 12+6 (EB) | - | - | - | - |  |  |
|  | ICH + vehicle | - | 12+6 (EB) | - | - | - | - |  |  |
|  | ICH + NaB (100mg/kg) | - | 12+6 (EB) | - | - | - | - |  |  |
|  | ICH + NaB (200mg/kg) | - | 12+6 (EB) | - | - | - | - |  |  |
| Exp.3 | Sham | 10 | - | - | - | - | - |  |  |
|  | ICH + vehicle | 10 | - | - | - | - | - |  |  |
|  | ICH + NaB (200mg/kg) | 10 | - | - | - | - | - |  |  |
| Exp. 4 | Sham | - | - | - | 3 | 6 | 5 |  |  |
|  | ICH + vehicle | - | - | - | 3 | 6 | 5 |  |  |
|  | ICH + NaB (200mg/kg) | - | - | - | 3 | 6 | 5 |  |  |
|  | ICH + NaB + MK2206 | - | - | - | - | 6 | 5 |  |  |
| Exp. 5 | Sham | - | - | 12 | - | 6 | - |  |  |
|  | ICH + NaB (200mg/kg) | - | - | 12 | - | 6 | - |  |  |
|  | ICH + NaB + scramble siRNA | - | - | 12 | - | 6 | - |  |  |
|  | ICH + NaB + DJ-1 siRNA | - | - | 12 | - | 6 | - |  |  |
| Exp. 6 | Sham | - | - | 12 | - | 6 | 5 |  |  |
|  | ICH + vehicle | - | - | 12 | - | 6 | 5 |  |  |
|  | ICH + NaB (200mg/kg) | - | - | 12 | - | 6 | 5 |  |  |
|  | ICH + NaB + rotenone | - | - | 12 | - | 6 | 5 |  |  |
|  | Subtotal | 30 | 90 | 96 | 9 | 114 | 44 | 12 |  |


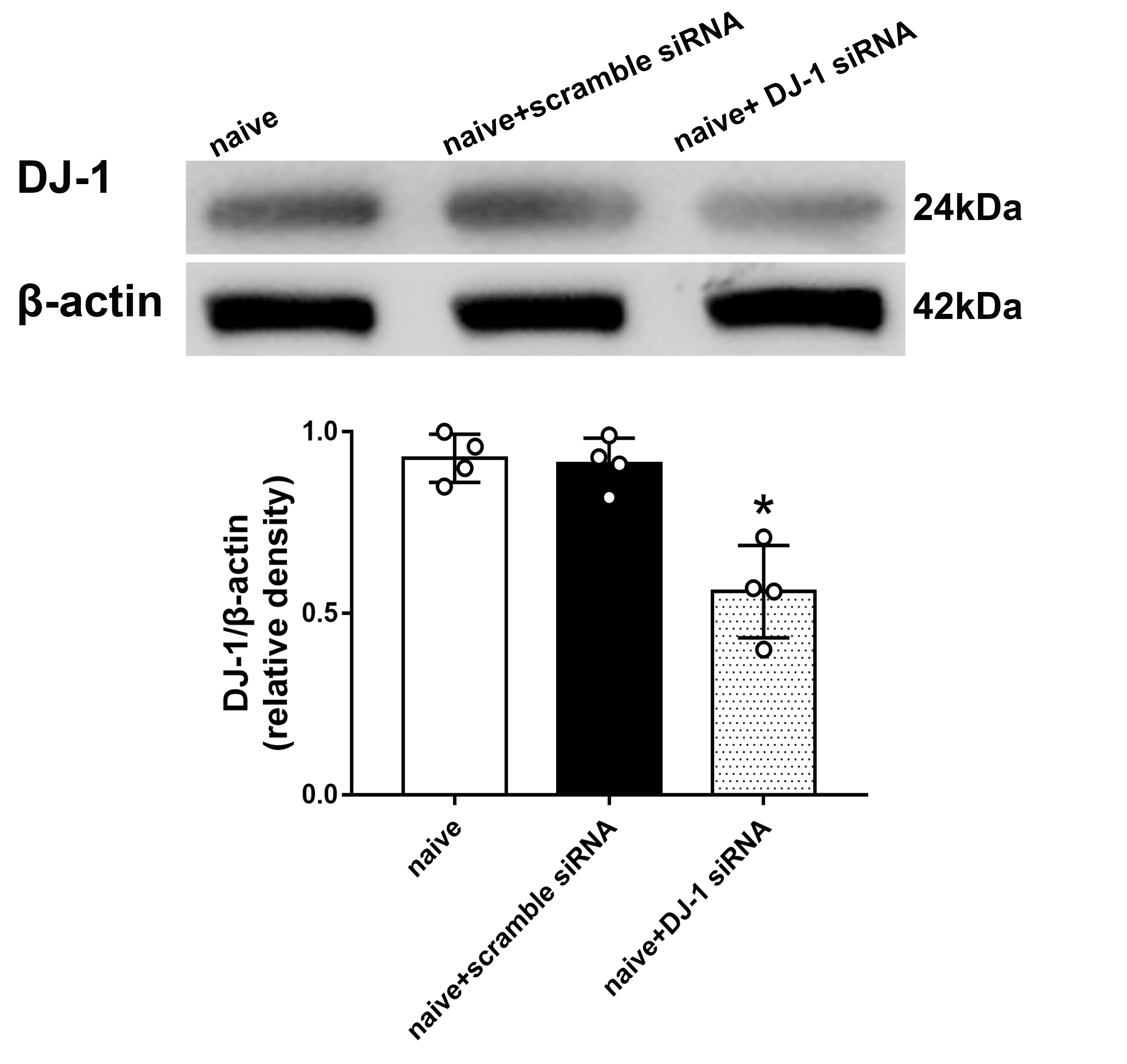


**Supplemental Fig. 1.** Depletion Efficiency of DJ-1 siRNA with Naïve Rats. n=4 for each group. The bars represent the mean ± SD. *p<0.05 versus naïve group.

**Supplemental Detailed procedures**

**ICH rat models**

Deep anesthesia was applied to the rats using pentobarbital (40 mg/kg, intraperitoneal injection). The operation was performed with the aid of a stereotaxic frame (Stoelting CO. USA). First, we isolated the right femoral artery and inserted with a polyethylene catheter (PE-160) to obtain blood for the following injection. Second, the skin on the top of the head was longitudinally incised with a scalpel. Third, we drilled a burr hole at the place 3.5 mm lateral right of the bregma. One hundred ul autologous blood, obtained from the right femoral artery, was manually injected into the right striatum (5.5 mm depth) using a Hamilton syringe with a 26 G needle. The needle was untouched for additional 10 minutes and then slowly withdrawn. Finally, the burr hole was closed with bone wax and the incision was sealed with sutures. For the sham group, rats received the same procedures except for the insertion of the needle.

**Morris water maze**

Rats were required to find the submerged platform, which was recorded by an overhead camera linked to a computer tracking system. We recorded their swim path, escape latency, and swim distance individually. The probe trial was performed on the last day of testing in which the platform was removed and the duration of time spent in the probe quadrant was recorded.

**IF staining**

After anesthetization, transcardial perfusion with 0.1 M PBS was performed, followed by another perfusion with 4 % paraformaldehyde (pH = 7.4). Then the cerebral hemispheres were removed and put into 4% PFA for post-fixation (4 °C, 24 h). After that, the brains were transferred to sucrose solution (30%, 2 days). Next, the brains were coronally sliced into 10mm sections, which were then fixed on slides and used for immunofluorescence staining, and then blocked with 5% normal donkey serum for 2 h at room temperature and incubated with primary antibodies at 4 °C overnight: DJ-1 (1:250, Abcam ab76008), caspase-3 (1:200, Abcam ab13847), NeuN (1:500, Abcam ab177487). After that, secondary antibodies were applied at room temperature for 2h. Finally, a fluorescence microscope (Olympus, Tokyo, Japan) was used to observe the sections and the photographs taken were post-processed with Photoshop 13.0 (Adobe Systems Inc., Seattle, WA). In addition, we used TUNEL (Roche Inc., Basel, Switzerland) and caspase-3 staining to quantitatively evaluate the cell apoptosis. Neuronal apoptosis was assessed by the proportion of TUNEL and caspase-3 positive cells in six sections at ×200 magnification of each brain sample. The results were showed as cells per square millimeter.

**Measurement of ROS Level**

The level of ROS in brain tissues was evaluated using a ROS assay kit (JianCheng, China). In brief, the samples were lysed in 0.01 mol/L PBS with centrifugation at 4­500g for 10 min. Then, the supernatant was extracted for the ROS assay. The supernatant (190 μl) and DCFH-DA (10 μl, 1 mol/L) were mixed in a micro-well at room temperature for 30 min. Afterwards, the mixtures were measured by fluorophotometry. Then the protein levels of different samples were acquired using a detergent-compatible protein assay kit (Bio-Rad, Hercules, CA, USA). Ultimately, the ROS levels were displayed in the form of fluorescence/mg protein.

**Measurement of ATP Levels**

As described in the instruction, the brain tissues were lysed in lysis buffer with centrifugation at 4 ℃ 12000g for 5 min. Then, the supernatant was extracted for the ATP assay. Before the assay, the ATP working reagents (100 μl; ATP detection reagent: ATP detection reagent diluent (1:9)) were added into a micro-well for 5 min at 37 °C. The samples (20 μl) were added and then (2s later at least) measured by Varioskan Flash (Thermo Fisher Scientific). The ATP concentrations were calculated through the standard curve method. Then the protein levels of different samples were acquired using a detergent-compatible protein assay kit (Bio-Rad, Hercules, CA, USA). Ultimately, the ATP levels were displayed in the form of nmol/mg.

**Transmission** **electron microscopy**

The rats received transcardial perfusion with 0.1 M PBS and 4 % paraformaldehyde (pH = 7.4) after anesthetization. Then the perihematomal tissues were collected and grained into 1 mm^3^ slices. After that, the slices were immersed into glutaraldehyde (2.5%) at 4°C overnight. Next, we put the samples in 1% osmium tetroxide for 1 h and dehydrated the samples with a series of graded ethanol. Then the tissues were immersed into a mixture of propylene oxide and resin (1:1). Four hours later, the samples were imbedded in resin. After that, we cut the samples in to 100nm sections and stained the sections with 4% uranyl acetate (20 min) and 0.5% lead citrate (5 min). Finally, the transmission electron microscopy (Philiphs Tecnai 10) was used to observe the ultrastructure of brain tissues.
